# Male Infertility Workup Needs Additional Testing of Expressed Prostatic Secretion and/or Post-Massage Urine

**DOI:** 10.1371/journal.pone.0082776

**Published:** 2013-12-09

**Authors:** Margus Punab, Tiiu Kullisaar, Reet Mändar

**Affiliations:** 1 Andrology Centre, Tartu University Hospital, Tartu, Estonia; 2 Department of Biochemistry, University of Tartu, Tartu, Estonia; 3 Department of Microbiology, University of Tartu, Tartu, Estonia; Northwestern University, United States of America

## Abstract

The male factor accounts for almost 50% of infertility cases. Inflammation may reduce semen quality via several pathways, including oxidative stress (OxS). As male infertility routinely is assessed using semen analysis only, the possible presence of non-leukocytospermic asymptomatic inflammatory prostatitis may be overlooked. We compared local and systemic OxS levels in male partners of infertile couples with different inflammation patterns in their genital tract and/or oligospermia.

Subjects (n=143) were grouped according to inflammation in their semen, expressed prostatic secretion (EPS), and/or post-massage urine (post-M). Systemic (8-isoprostanes in urine) and local (diene conjugates and total antioxidant capacity in seminal plasma) OxS was measured

The levels of OxS markers were significantly elevated in both severe inflammation groups – leukocytospermic men and subjects whose inflammation was limited only to EPS and/or post-M. Comparison between oligospermic and non-oligospermic men with genital tract inflammation, and oligozoospermic men with or without inflammation in the genital tract indicated that inflammation but not oligospermia status had significant impact on the measured OxS markers.

Hence, a high leukocyte count in prostate-specific materials (EPS, post-M), even in absence of clear leukocytopsermia, is an important source of local and systemic OxS that may be associated with male infertility and affect general health. We suggest including the tests for detection of inflammation of the prostate into the workup of infertile men as was suggested in the WHO 1993 recommendation.

## Introduction

Infertility represents an increasing medical problem affecting 15% of couples, and its treatment is stressful, invasive, and costly. The male factor accounts for almost 50% of infertility cases. Besides clearly defined causes of male infertility (genetic disorders, ductal obstruction, etc.), there are several possible causes where the exact mechanism of sperm dysfunction is not known yet, with genital tract inflammations and varicocele being among the most common ones [1].

Inflammation in the male genital tract may be associated with reduced semen quality via several pathways, including oxidative stress (OxS) associated with white blood cells – a shift of pro-oxidant/antioxidant balance towards a domination of oxidants. OxS develops when there is an imbalance between the generation of free radicals and the scavenging capacity of anti-oxidants in the reproductive tract. It may cause DNA damage in spermatozoa and affect sperm number, motility, and morphology, but also sperm-oocyte fusion. Elevated levels of reactive oxygen species (ROS) can be seen in 30–80% of men with male infertility [2–5].

Since the assessment of male infertility is routinely done using semen analysis, most of the studies published so far about the connection of male infertility and OxS are based on this analysis only. Therefore, the possible presence of non-leukocytospermic asymptomatic inflammatory prostatitis may have been overlooked in these studies.

We aimed to reveal the association of OxS in the male partners of infertile couples with different inflammation patterns in their genital tract and/or oligospermia.

## Materials and Methods

### Study Group

The study was carried out at the Andrology Centre of Tartu University Hospital from May 2009 to March 2010 and included 143 men who participated in a prospective study on male infertility.

Study subjects consulted an andrologist due to couple infertility (trying to conceive >1 year), while their partners were investigated for causes of infertility at the same time. Invitation to participate was given to subjects with oligozoospermia (sperm concentration <20 M/ml, total sperm count <40 M/ejaculate) and leukocytospermic men (>1 M WBC/ml). Subjects were grouped according to inflammation in their semen, expressed prostatic secretion (EPS), and/or post-massage urine (post-M) (Table 1). None of the subjects complained of lower urinary tract symptoms or genitourinary pain, therefore NIH IV category (asymptomatic inflammatory) prostatitis [6] was diagnosed in all men with inflammation.

**Table 1:** Clinical and oxidative stress parameters of the study subjects.

	Group I (n=29)	Group II (n=31)	Group III (n=24)	Group IV (n=32)	Group V (n=27)	P values *
	Severe inflammation in semen	Severe inflammation in EPS and/or post-M	Mild inflammation in semen, EPS and/or post-M	Oligospermia without inflammation	Controls	
Age (y)	33.6 ± 6.2	31.0 ± 7.8	31.7 ± 5.2	32.2 ± 6.6	30.0 ± 5.2	NS
Period of abstinence (d)	3.7 ± 1.4	3.9 ± 1.7	3.2 ± 0.9 ^1^	3.9 ± 1.5	5.9 ± 6.7 ^1^	p=0.024
Total sperm count (M)	57.8 ± 51.8 ^1^	41.2± 45.6 ^2^	36.8 ± 53.1 ^3^	12.6 ± 26.3 ^4^	446.7 ± 295.8 ^1-4^	p<0.001
Semen volume (ml)	4.4 ± 1.6	4.5 ± 1.5	4.1 ± 1.5	4.1 ± 1.5	4.4 ± 1.7	NS
Motility A+B (%)	37.3 ± 17.4 ^1^	34.3 ± 14.3 ^2^	36.1 ± 18.3 ^3^	27.0 ± 19.0 ^4^	50.0 ± 11.5 ^1-4^	p<0.001
Morphologically normal spermatozoa (%)	6.0 ± 4.7 ^1^	5.6 ±4.1 ^2^	5.5 ± 4.1 ^3^	4.1 ± 3.4 ^4^	12.7 ± 7.0 ^1-4^	p<0.001
WBCs in semen (M/ml)	2.7 ± 1.9 ^1-4^	0.2 ± 0.2 ^1^	0.3 ± 0.2 ^2^	0.0 ± 0.1 ^3^	0.0 ± 0.1 ^4^	p<0.001
IL-6 in semen (pg/ml)	154 ± 161 ^1-4^	39 ± 41 ^1^	28 ± 18 ^2^	22 ± 15 ^3^	20 ± 12 ^4^	p<0.001
WBCs in EPS (M/ml)	2.3 ± 3.3	4.5 ± 5.5 ^1,2^	0.4 ± 0.3 ^1^	0.0 ± 0.1 ^2^	nd	p<0.001
WBCs in post-M by cytology (WBC per HPF visual field)	6.7 ± 7.3 ^1^	12.6 ± 11.7 ^1,2,3^	0.8 ± 0.8 ^2^	0.7 ± 0.8 ^3^	nd	p<0.001
WBCs in post-M by haemocytometer (WBC/mm^3^)	98.2 ± 172.2 ^1,2^	69.3 ± 123.1 ^3,4^	0.1 ± 0.3 ^1,3^	0.3 ± 0.6 ^2,4^	nd	p=0.002
IL-6 in EPS (pg/ml)	209 ± 223 ^1,2^	283 ± 784 ^3,4^	45 ± 58 ^1,3^	37 ± 16 ^2,4^	nd	p=0.002
DC in seminal plasma (μM)	11.6 ± 6.0 ^1,2^	9.9 ± 7.4 ^3,4^	9.2 ± 7.5	5.5 ± 4.1 ^1,3^	5.1 ± 2.0 ^2,4^	p<0.001
ROS-TAC score	5.9 ± 4.1 ^1-3^	4.5 ± 3.5 ^4,5^	3.5 ± 2.9 ^1^	2.3 ± 1.6 ^2,4^	1.8 ± 0.9 ^3,5^	p<0.001
8-isoprostanes in urine (ng/mmol creatinine)	86.7 ± 26.3 ^1-3^	69.9 ± 34.9 ^4,5^	51.1 ± 27.2 ^1^	49.3 ± 21.6 ^2,4^	39.3 ± 14.8 ^3,5^	p<0.001

Plus–minus values are means ±SD.

NS – not significant, nd – not detected, EPS – expressed prostatic secretion, WBC – white blood cells, post-M – post-massage urine, IL-6 - interleukin-6, DC – diene conjugates, TAC – total anti-oxidant capacity, ROS – reactive oxygen species, ROS-TAC score – ratio of DC and TAC.

* One-Way ANOVA; post-hoc Bonferroni between each pair of the similarly superscripted data at the same row p<0.05.

Group I (n=29) contained men with severe inflammation in semen (>1 M WBC/ml), irrespective of inflammatory status of the prostate-specific materials. Among them 17 subjects showed severe (>1 M WBC/ml in EPS and/or >10 WBC/mm^3^ in post-M), 6 showed borderline (0.5-0.9 M WBC/ml in EPS and/or 5-9 WBC/mm^3^ in post-M), and 6 showed missing inflammatory reaction (<0,5 M WBC/ml in EPS and/or <5 WBC/mm^3^ in post-M) in prostate-specific materials. Seven men were oligozoospermic.

Group II (n=31) contained men with borderline (0,2-0,99 M WBC/ml, n=20) or missing (<0,2 M WBC/ml, n=11) leukocytospermia, but with severe inflammation in EPS and/or post-M. Seventeen men were oligozoospermic.

Group III (n=24) contained men with borderline inflammation in semen (n=20) and/or EPS (n=9). No WBSs in post-M were detected in these men. Thirteen men were oligozoospermic.

Group IV men (n=32) had oligozoospermia with no inflammation in semen, EPS, or post-M.

The control group (Group V, n=27) was formed of asymptomatic inflammation-free fertile men (partners of pregnant women).

Exclusion criteria were stated according to suggestions of the NIH workshop on chronic prostatitis in Bethesda, 1995 [7]. None of these men had received anti-microbial therapy within 3 months and anti-inflammatory medications for at least 1 month before evaluations. We excluded all subjects with signs suggestive of urethritis and/or balanoposthitis, as well as those positive for some sexually transmitted disease.

### Ethics statement

Participation in the study was voluntary. All subjects were at least 18 years old. Informed consent was obtained from all study subjects. The study was approved by the Ethics Review Committee on Human Research of Tartu University, Estonia.

### Specimens

Semen samples were collected by patients in a private room near the laboratories after they washed their glans penis with soap and water. Semen samples were obtained after urinating by masturbation and were ejaculated into sterile collection tubes. The samples were then incubated at 37° C for 25–45 min for liquefaction, and thereafter the seminal plasma was separated by centrifugation.

Four to five days after semen analysis, EPS was obtained by digital massage (M) approximately 5 min after voiding (pre-M). After digital massage, subjects were told to collect the first 20 ml of urine (post-M).

For biochemical analyses, the urine and seminal plasma were frozen at -20° C. Levels of 8-isoprostanes (8-EPI) were measured in the urine; all other OxS parameters were measured in the seminal plasma.

### Basic semen parameters

The analysis of semen was performed according to WHO guidelines [8]. Semen volume was estimated by weighing the collection tube with the semen sample and subsequently subtracting the pre-determined weight of the empty tube (assuming 1 g = 1 ml). Motility was assessed to report the number of motile spermatozoa (WHO motility classes A + B). Sperm concentration was assessed using improved Neubauer haemocytometer. Total sperm count was calculated by multiplying semen volume by sperm concentration.

### Cytological analysis of semen

Semen smears were made for detecting the WBC count. The smears were air dried, Bryan–Leishman stained, and examined with the use of oil immersion microscopy (magnification: x1000) by an experienced microscopist. The WBC concentration in semen was calculated by using known sperm parameters (as 10^6^ ml^-1^), according to following formula:

[WBC] = Number of WBCs counted / Number of spermatozoa counted x Semen sperm concentration

One hundred round cells were counted twice, and their mean value was registered.

### EPS processing

Processing of EPS started within 20 minutes after collection. EPS analysis was performed as recommended according to WHO guidelines for semen analysis [8].

### Round cell count in EPS

Each wet mount was made by placing 10 μl of well-mixed EPS onto a glass slide. Cover glass of 22 mm was used. Examination with phase contrast optics at 400x magnification of the wet preparation began as soon as the “flow” in the preparation had ceased. All round cells were recorded from at least 5 visual fields. The wet preparation was used to estimate approximate concentration [4 round cells per field approximately correspond to a concentration of 1 M/ml) and to select the most appropriate dilution for hemocytometer analysis. Standard dilutions were 1+4 and 1+1. An exact volume of fluid was withdrawn from a well-mixed EPS sample with a positive displacement pipette and added to diluent in a test tube with a tight lid.

Tubes containing diluted samples were mixed for at least 10 seconds (in a vortex mixer) immediately before filling the counting chamber. After mixing, an aliquot of about 6–10 μL was added with a pipette to one side of an improved Neubauer haemocytometer. Then a second aliquot was placed on the other side. The filled chamber was left to rest for 10–15 minutes in a humid box to allow the round cells to sediment to the grid of the counting chamber. All round cells were counted in the entire 25-square central grid on both sides of chamber. If the difference between two counts was equal to or less than a 10% average, the cell counts were corrected for dilution factor. If not, the counts were discarded and new assessments starting from dilution were made.

### WBC and neutrophil counts in EPS

Smears were made from wet mount slides (using 10 μl of EPS), air dried, Bryan–Leishman stained and examined using an oil immersion microscope by an experienced andrology lab technician. The percentage of WBCs from all round cells was counted. Whenever possible, one hundred round cells were counted twice and the mean value was registered. The neutrophil concentration (M/ml) was calculated using a known concentration of round cells multiplied by the neutrophil percentage from the cytological analysis and divided by 100.

One technician performed the assessments of all semen and EPS samples during the course of the study.

### Urine analysis

Both pre-M and post-M urine samples were sent for analysis. Pre-M was used to exclude subjects with urethritis and cystitis.

The WBCs in urine were first counted using a Urisys 1100 Urine Analyzer (Roche Diagnostics Corporation, Basel, Switzerland). In addition to automated analysis, the sediments of urine were analyzed. Modified Steinheimer supravital staining was used to ease identification of leukocytes. 10 ml of urine was centrifuged for 10 minutes at 200 g. The supernatant was poured off, and 50 μl of Reastain urine stain (Reagena, Finland) was added to the remaining pellet (0,5 ml). Then 13 μl of stained sediment was transferred to a microscopic slide and covered with cover glass of 18x18. During examination with phase contrast optics at 400x magnification, round cells from at least 10 visual fields were recorded and the average number was calculated.

### Detection of IL-6 in seminal plasma and EPS

Interleukin-6 levels of seminal plasma and EPS were measured using an Immulite Automated Chemiluminescence Immunoassay Analyzer (Immulite DPC, Los Angeles, CA, USA) according to the manufacturer’s instructions. If the secretion volume was less than 20 μl, a known volume of prostate secretion plasma was diluted according to manufacturer instructions. Any dilution factor was recorded, and results were corrected appropriately.

### Detection of oxidative stress markers

Detection of diene conjugates (DC) and total antioxidant capacity (TAC) in seminal plasma and 8-Isoprostanes (8-EPI) in urine is described elsewhere [9]. The ROS-TAC score was calculated as the ratio of DC and TAC.

### Statistical methods

For statistical analyses, SigmaStat (Systat Software, Chicago, IL, USA) software was used. The differences between the five groups were analyzed using one-way analysis of variance (ANOVA), followed by Bonferroni post hoc test. The two-way ANOVA was used to examine the effect of inflammation and oligospermia on oxidative stress parameters. A principal coordinate analysis performed in PAST was used to visualize similarities between samples. The Spearman correlation was used to determine correlations between the parameters. Statistical significance was assumed at *p* < 0.05 for all parameters.

## Results

The clinical data of study subjects are presented in Table 1. No differences were found between the groups regarding age. The results of basic semen parameters followed the inclusion criteria- with the best in the control group and the worst in oligospermic men. Differences in sperm count arise from the selection process. Semen volume, spermatozoa motility, and percentage of normal spermatozoa were similar in the three inflammation groups.

Markers of inflammation were used to define the study groups (Table 1). Semen IL-6 counts and WBC counts were in good correlation (R=0.64, p<0.001), and also EPS IL-6 counts were in good correlation with WBC counts both in EPS (R=0.68, p<0.001) and post-M (R=0.54, p<0.001).

**Figure 1:**
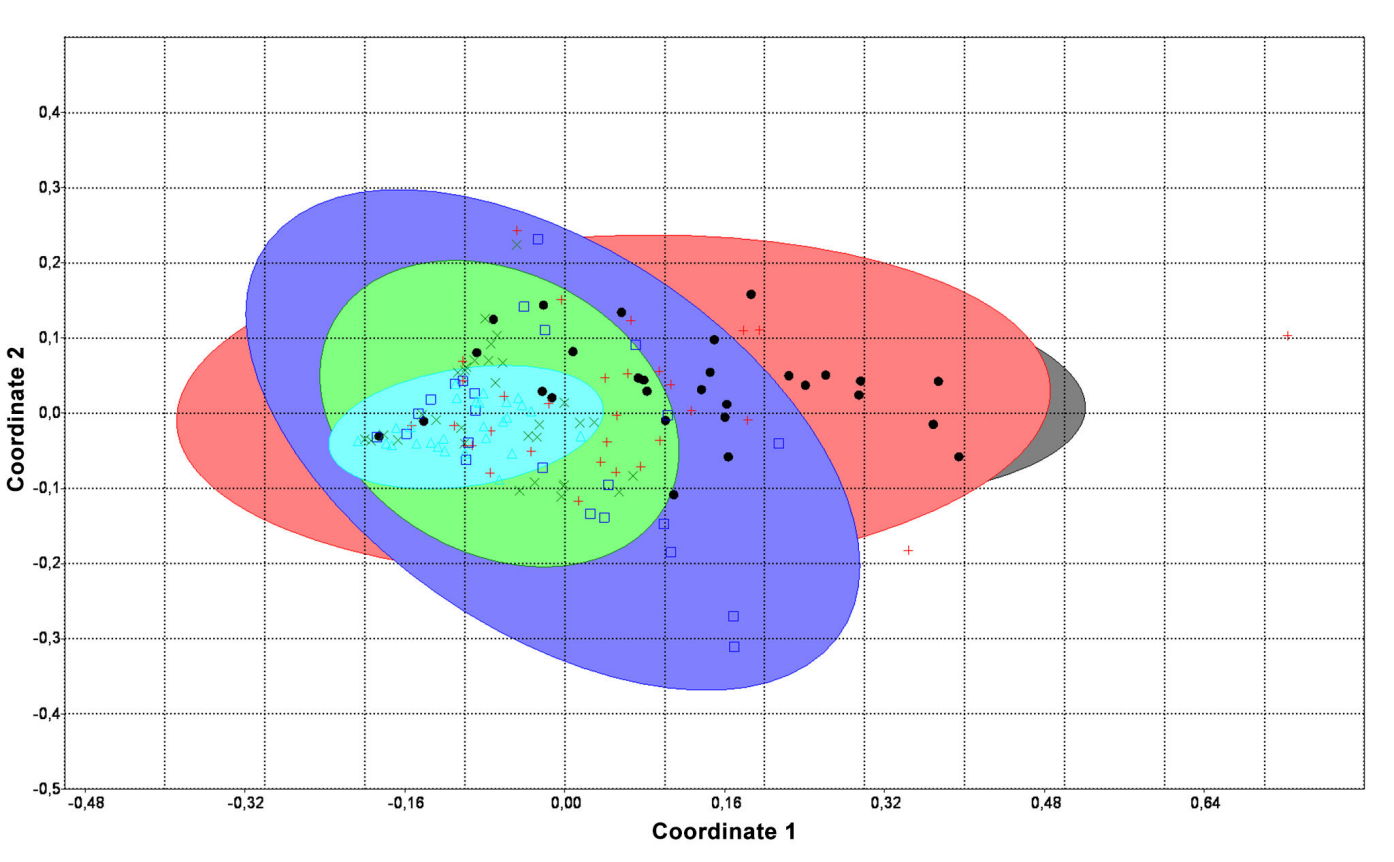
Two-dimensional plot of the Principal Coordinate Analysis (PCoA). The figures shows the clustering of of OxS data (DC, ROS-TAC score and 8-isoprostanes) and illustrating high similarity of both severe inflammation groups. Black dot, Group I; red cross, Group II, violet square, Group III; green cross, Group IV; blue triangle, Group V.

The levels of OxS markers (8-isoprostanes, DC, ROS-TAC score) were clearly elevated in both severe inflammation groups — leukocytospermic men (Group I) and subjects whose inflammation was limited only to prostate-specific material (EPS and/or post-M, Group II) (Table 1). A similar grouping pattern for Groups I and II is revealed also on the PCoA plot (Figure 1).

As there were oligospermic subgroups in all three inflammation groups, we performed additional analysis to reveal the effect of inflammation and oligospermia on OxS parameters. The two-way ANOVA detected an inflammation effect, but did not detect an oligospermia effect (except a marginal effect for isoprostanes) or an inflammation by oligospermia interaction (Table 2).

**Table 2:** Effect of inflammation and oligospermia on OxS parameters.

	Severe inflammation in semen, EPS and/or post-M (Groups I and II, n=60)		Mild or missing inflammation in semen, EPS and/or post-M (Groups III, IV and V, n=83)		Effect of inflamma-tion (*P* value)	Effect of oligo-spermia (*P* value)	Inter-action (*P* value)
	Oligo-spermia (n=24)	Normal sperm count (n=36)	Oligo-spermia (n=45)	Normal sperm count (n=38)			
DC in seminal plasma (μM)	11.2 ± 7.9	10.3 ± 5.9	6.9 ± 5.9	6.0 ± 4.0	<0.001	0.158	0.845
ROS-TAC score	5.0 ± 3.8	5.3 ± 3.9	2.7 ± 2.2	2.2 ± 1.7	<0.001	0.504	0.950
8-isoprostanes in urine (ng/mmol creatinine)	81.4 ± 36.6	75.8 ± 28.8	50.7 ± 25.1	41.7 ± 16.4	<0.001	0.043	0.921

Plus–minus values are means ±SD.

EPS – expressed prostatic secretion, post-M – post-massage urine, DC – diene conjugates, TAC – total anti-oxidant capacity, ROS – reactive oxygen species, ROS-TAC score – ratio of DC and TAC.

The data were analyzed by two-way ANOVA.

## Discussion

Our study revealed that inflammation in prostate-specific materials (EPS and/or post-M), even in the case of a low number of leukocytes in semen, is an important source of local (seminal) and systemic oxidative stress. If we accept OxS as causative or a co-factor of male infertility/subfertility, this finding disconfirms the common misconception that only semen should be investigated in infertile males.

Prostatitis is not widely accepted as a precursor or cause of infertility since several studies had revealed no association between leukocytes in prostatic secretions and reduced standard semen parameters [10,11]. This finding (probably together with a shortage of clinical andrologists) may be why previously recommended [12] investigations of prostate-specific materials were forgotten. At the same time, male infertility is frequently associated with low level leukocyte infiltration in semen [13–16], and some studies have shown that antimicrobial treatment in the case of low level leukocytospermia may increase pregnancy rates [17].

We revealed that a high number of leukocytes in EPS and/or post-M may occur also in the case of minimal or missing leukocytes in semen. In our previous study, we showed that the currently suggested limit of inflammation for semen samples (>1 M WBC/ml) may be too high to discriminate patients who need to be treated for genital tract inflammation [18]. Our present study, where more prostate specific specimens (EPS, post-M) were additionally investigated, confirmed this idea. The present study included 40 subjects whose semen contained 0,2–0,99 M WBC/ml and 47 subjects with no leukocytospermia (excluding controls), and we detected severe inflammatory reactions in their prostate in 50% and 23% of cases, respectively.

In addition to inflammation markers, we also measured OxS markers in these men. In the case of chronic inflammation, the WBCs appear as the main source of ROS [19]. Besides the local OxS markers that were detected in the seminal plasma, we measured 8-isoprostanes in urine for detecting systemic OxS as suggested by the Society of Free Radical Research [20]. We found significant elevation of these markers in both severe inflammation groups, and a tendency for these markers to increase among subjects with a mild inflammatory reaction. Our previous studies [21–23] have revealed that OxS is an important pathogenesis mechanism in several forms of chronic prostatitis and that significant positive correlation exists between 8-isoprostanes with 8-OHdG (8-hydroxy-2’-deoxyguanosine) content indicating the oxidative damage of DNA. In a recent publication, DNA fragmentation (measured by TUNEL) and oxidative DNA damage (measured by 8-OHdG levels) were found to be highly correlated [24]. Future studies should confirm a direct connection between sperm functional parameters and isolated prostate inflammation.

Moreover, experimental and clinical studies have revealed that OxS may play an etiological role in the development and progression of many chronic inflammatory and degenerative diseases, including non-inflammatory conditions such as cardiovascular diseases [25,26]. Our study indicated that asymptomatic chronic inflammation in the male genital tract may be an important source of systemic OxS. It is important to know that this effect is not limited solely to men but affects also their sexual partners as was indicated in our latest paper [9]. Since there is only very limited information available about links between chronic inflammatory diseases of the prostate and the general health of men, then future studies on this issue are urgently needed.

Although these results may lead to the idea that empiric antioxidant therapy could be favorable in men with unexplained infertility, one should bear in mind that a certain limited amount of ROS is essential in order to trigger vital physiological reactions in cells, including capacitation or the acrosome reaction in sperm. Therefore, unjustified antioxidant treatment might derail the system towards reduced status, which is unphysiological and can even induce cancer. This is called the ‘antioxidant paradox’ [27]. Therefore, a proper andrological diagnostic work-up has to be carried out beforehand.

## Conclusions

A high leukocyte count in prostate-specific materials (EPS, post-M), even in the absence of clear leukocytopsermia, is an important source of seminal and systemic OxS that may be associated with male infertility. Therefore, we suggest including the tests for detection of inflammation status of the prostate into the workup of infertile men as was suggested in the WHO 1993 recommendation. In addition, there is an urgent need for studies on the effects of chronic inflammatory diseases of the prostate on the general health of men.
